# Mitral annulus disjunction in consecutive patients undergoing cardiovascular magnetic resonance: Where is the boundary between normality and disease?

**DOI:** 10.1016/j.jocmr.2024.101056

**Published:** 2024-07-04

**Authors:** Stefano Figliozzi, Kamil Stankowski, Lara Tondi, Federica Catapano, Mauro Gitto, Costanza Lisi, Sara Bombace, Marzia Olivieri, Francesco Cannata, Fabio Fazzari, Renato Maria Bragato, Georgios Georgiopoulos, Pier-Giorgio Masci, Lorenzo Monti, Gianluigi Condorelli, Marco Francone

**Affiliations:** a IRCCS Humanitas Research Hospital, Via Alessandro Manzoni, 56, Rozzano, Milano, Italy; b Department of Biomedical Sciences, Humanitas University, Via Alessandro Manzoni, 56, Pieve Emanuele, Milano, Italy; c Multimodality Cardiac Imaging Section, IRCCS Policlinico San Donato, San Donato Milanese, Milano, Italy; d Department of Clinical Therapeutics, National and Kapodistrian University of Athens, Athens, Greece; e School of Biomedical Engineering and Imaging Sciences-Faculty of Life Sciences and Medicine, King's College London, London, UK

**Keywords:** Mitral annulus disjunction, Prevalence, Mitral valve prolapse, Cardiac magnetic resonance, Sudden cardiac death

## Abstract

**Background:**

The presence of mitral annulus disjunction (MAD) has been considered a high-risk feature for sudden cardiac death based on selected study populations. We aimed to assess the prevalence of MAD in consecutive patients undergoing clinically indicated cardiovascular magnetic resonance (CMR), its association with ventricular arrhythmias, mitral valve prolapse (MVP), and other CMR features.

**Methods:**

This single-center retrospective study included consecutive patients referred to CMR at our institution between June 2021 and November 2021. MAD was defined as a ≥1 mm displacement between the left atrial wall-mitral valve leaflet junction and the left ventricular wall during end-systole. MAD extent was defined as the maximum longitudinal displacement. Associates of MAD were evaluated at univariable and multivariable regression analysis. The study endpoint, a composite of (aborted) sudden cardiac death, unexplained syncope, and sustained ventricular tachycardia, was evaluated at a 12-month follow-up.

**Results:**

Four hundred and forty-one patients 55 ± 18 years, 267/441 (61%) males) were included, and 29/441 (7%) had MVP. The prevalence of MAD ≥1 mm, 4 mm, and 6 mm was 214/441 (49%), 63/441 (14%), and 15/441 (3%), respectively. Patients with MVP showed a higher prevalence of MAD greater than 1 mm (26/29 (90%) vs 118/412 (46%)); p < 0.001), 4 mm (14/29 (48%) vs 49/412 (12%)); p < 0.001), and 6 mm (3/29 (10%) vs 12/412 (3%)); p = 0.03), and a greater MAD extent (4.2 mm, 3.0–5.7 mm vs 2.8 mm, 1.9–4.0 mm; p < 0.001) compared to patients without MVP. MVP was the only morpho-functional abnormality associated with MAD at multivariable analysis (p < 0.001). A high burden of ventricular ectopic beats at baseline Holter-electrocardiogram was associated with MAD ≥4 mm and MAD extent (p < 0.05). The presence of MAD ≥1 mm (0.9% vs 1.8%; p = 0.46), MAD ≥4 mm (1.6% vs 1.3%; p = 0.87), or MVP (3.5% vs 1.2%; p = 0.32) were not associated with the study endpoint, whereas patients with MAD ≥6 mm showed a trend toward a higher likelihood of the study endpoint (6.7% vs 1.2%; p = 0.07).

**Conclusion:**

MAD of limited severity was common in consecutive patients undergoing CMR. Patients with MVP showed higher prevalence and greater extent of MAD. Extended MAD was rarer and showed association with ventricular arrhythmias at baseline. The mid-term prognosis of MAD seems benign; however, prospective studies are warranted to search for potential “malignant MAD extents” to improve patients’ risk stratification.

## Graphical abstract

**Figure:**
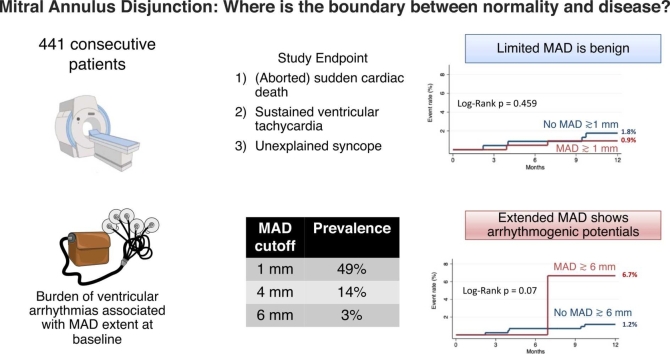

## 1 Background

Mitral annulus disjunction (MAD) is a displacement between the atrial wall-mitral valve leaflet junction and the left ventricular (LV) myocardial attachment [1], [2], [3], [4], [5], [6], [7]. The presence of MAD was initially suggested to represent a benign anatomical variant of the mitral apparatus [8] but has been recently considered a high-risk feature of sudden cardiac death (SCD) [5], [6], [9], [10]. The association between the presence of MAD and malignant ventricular arrhythmias in patients with arrhythmic mitral valve prolapse (MVP) [2], [3], [5], [6], as well as in patients without MVP [11], has increased interest in this imaging parameter. However, MAD assessment has been mainly limited to selected cohorts of patients with MVP and/or arrhythmic presentation [2], [3], [4], [11], whereas its prevalence in consecutive patients remains unknown. Cardiovascular magnetic resonance (CMR) is the ideal imaging modality to detect this condition [12]. The present study aimed to evaluate the prevalence of MAD in consecutive patients clinically referred to CMR, its association with ventricular arrhythmias, MVP, and other CMR features.

## 2 Methods

### 2.1 Study design

This was a single-center retrospective study of prospectively collected data including consecutive patients clinically referred to CMR at our laboratory (IRCCS Humanitas Research Hospital, Milan, Italy) between June 2021 and November 2021. The study inclusion criteria were i) absence of contraindication to CMR, ii) feasibility of MAD assessment at CMR study, and iii) feasibility of clinical follow-up. All patients provided written informed consent and the institutional review board approved the study protocol. Patients also underwent clinical visits on CMR date.

Baseline ventricular arrhythmias were evaluated in patients with available electrocardiogram (ECG) monitoring at presentation and included ventricular ectopic beats (VEBs) ≥10,000/day, non-sustained ventricular tachycardias (i.e., ≥3 consecutive ventricular beats at a rate of ≥100 bpm), sustained ventricular tachycardias (i.e., lasting ≥30 s), and ventricular fibrillation. The study endpoint was a combination of (aborted) SCD, unexplained syncope, and sustained ventricular tachycardia at 1-year follow-up. Clinical follow-up was performed through clinical visits, telephonic interviews, and interrogation of electronic health records.

### 2.2 CMR acquisition and analysis

CMR scans were acquired using a 1.5T scanner (Siemens AERA, Siemens Healthineers, Erlangen, Germany). A standardized protocol was carried out including i) cine images in 2-chamber, 3-chamber, and 4-chamber views; ii) stack of short-axis cine images covering both ventricles; iii) native T1/T2-mapping analysis and LGE (late gadolinium enhancement) images in the same orientation of cine images [13]. Images were analyzed through a Circle CVI42 station-version-5.13.7 (Circle Cardiovascular Imaging Inc., Calgary, Alberta, Canada) according to current recommendations [14], [15]. An experienced operator (S.F., level 3 certificate in CMR, European Society of Cardiology) blinded to demographic, clinical, and CMR data searched for MVP and MAD in standard long-axis views (2-chamber, 3-chamber, and 4-chamber views). MVP was defined as a systolic displacement ≥2.0 mm of one or both mitral valve leaflets above the annulus in 3-chamber long-axis view [16], [17]. Mitral annulus disjunction was defined as a separation ≥1.0 mm between the left atrial wall-mitral valve leaflet junction and the basal LV wall during end-systole [11], [18]. MAD extent was defined as the maximum longitudinal displacement in any long-axis view [2], [4], [11]. In 3-chamber and 4-chamber views, the basal LV septal wall was excluded by MAD analysis due to the presence of the mitro-aortic curtain and the absence of left atrial wall above the myocardium [6], [11]. Patients with inadequate images precluding MAD analysis in one or more long-axis views were excluded. In patients with qualitative evidence of mitral regurgitation, phase contrast sequences transaxial to the ascending aorta were performed to quantify the regurgitation through the indirect method. Mitral regurgitation was defined as more than mild in the presence of a regurgitant volume or fraction, respectively, ≥30 mL and 30% [19], [20]. Mitral annulus antero-posterior diameter was measured during end-diastole and end-systole at 3-chamber long-axis cine views [2], [21]. Patients without abnormalities at morpho-functional assessment or tissue characterization were defined as “normal” CMR exams. Details on CMR acquisition protocol are available in the Supplementary Material.

### 2.3 Reproducibility analysis

The intra-observer reproducibility of MAD measurements was tested by re-analyzing 20 random datasets 2 weeks apart by the same researcher (S.F.) blinded from the initial measurements. The inter-observer variability was tested by having the same datasets analyzed by a different expert researcher (F.C., 5 years of experience in CMR) who was not aware of the results of the other observer.

### 2.4 Echocardiography subgroup

In 16 patients, transthoracic echocardiography (TTE) imaging was available and was used to analyze the presence and extent of MAD by an expert researcher (S.F., 6 years of experience in third-level echocardiography laboratories), blinded from the CMR measurements.

### 2.5 Statistical analysis

The Kolmogorov-Smirnov test was used to check the variables’ distribution. Continuous variables were expressed as mean ± standard deviation or median (25th/75th percentiles) as appropriate. Categorical variables were reported as numbers and percentages. Continuous variables were compared by means of the independent Student’s t-test, Mann-Whitney, and analysis of variance, as appropriate, while categorical data by means of chi-square test. Intra- and inter-observer reproducibility of MAD measurements was evaluated by using two-way mixed intra-class correlation coefficient (ICC), Bland-Altman analysis, Pearson correlation coefficient (r), and Cohen's kappa coefficient. Associates of MAD presence and extent, including the baseline arrhythmic burden in patients with available baseline Holter-ECG monitoring, were tested by univariable and multivariable linear and logistic regression. Significant variables at univariable analysis were selected for multivariable analysis. Survival curves for the composite endpoint were constructed with the use of Kaplan-Meier estimates and compared with the log-rank test. Firth and Poisson regression were used as sensitivity analyses given the low number of expected events. Patients who experienced more than one event were censored at the time of the first event. Data analysis was performed using R (the R Foundation for Statistical Computing, version 4.1.2) and Stata, version 17 (Stata Corp, College Station, Texas, USA). All reported p values were two-sided and p < 0.05 was considered to be statistically significant.

## 3 Results

### 3.1 Study population

**Fig. 1:**
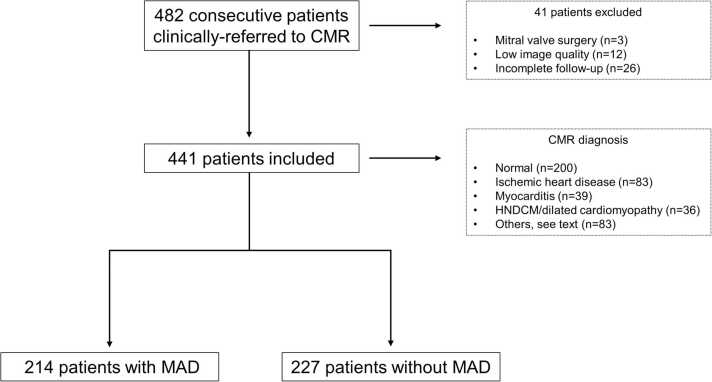
Study flowchart. *CMR* cardiovascular magnetic resonance, *HNDCM* hypokinetic non-dilated cardiomyopathy, *MAD* mitral annulus disjunction.

**Fig. 2:**
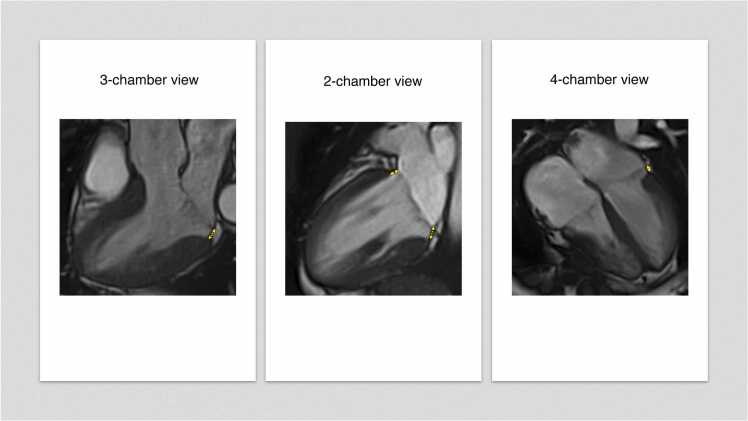
Mitral annulus disjunction in standard long-axis CMR cine views. Mitral annulus disjunction (yellow arrows) is evident in standard long-axis cine views during end-systole. *CMR* cardiovascular magnetic resonance.

We screened 482 patients clinically referred to CMR at our institution from June 2021 to November 2021. Twelve and three patients were, respectively, excluded because of unfeasible MAD analysis secondary to inadequate image quality and previous mitral valve surgery. Twenty-six patients were lost to follow-up and excluded from the analysis. The final study population consisted of 441 patients (267 men, 61%; age: 54.8 ± 17.8 years) (Figs. 1 and 2). One hundred and forty out of four hundred and forty-one patients (32%) had 24-hour Holter-ECG available at baseline presentation. The most common CMR diagnoses were “normal heart” in 200/441 (45%) patients and ischemic heart disease in 83/441 (19%). Twenty-nine/441 (7%) patients presented with MVP. Demographic, clinical, and CMR characteristics stratified by MAD ≥1 mm, 4 mm, and 6 mm are summarized in Table 1, Supplementary Table 1, and Supplementary Table 2**,** respectively.

**Table 1:** Clinical and CMR characteristics of the study population according to MAD presence.

Variables	MAD absence (n = 227)	MAD presence (n = 214)	p value
*Demographic and clinical data*			
Age, years	57 (47-71)	55 (41-67)	**0.04**
Female, n	85/227 (38%)	89/214 (42%)	0.37
Athletes, n	13/227 (6%)	5/214 (2%)	0.06
Previous PCI, n	50/227 (22%)	36/214 (17%)	0.30
Hypertension, n	89/227 (39%)	75/214 (35%)	0.38
Diabetes, n	27/227 (12%)	19/214 (9%)	0.26
Hypercholesterolemia, n	75/227 (33%)	73/214 (34%)	0.81
No symptoms, n	125/227 (55%)	132/214 (62%)	0.16
Typical chest pain, n	10/227 (4%)	12/214 (6%)	0.45
Atypical chest pain, n	26/227 (11%)	14/214 (7%)	0.07
Palpitations, n	17/227 (8%)	18/214 (8%)	0.72
Dyspnea, n	48/227 (21%)	40/214 (19%)	0.52
Unexplained syncope, n	4/227 (2%)	1/214 (1%)	0.20
24-hour Holter-ECG, n	63/227 (28%)	77/214 (36%)	0.06
VEBs ≥10,000 per day, n	2/63 (3%)	6/77 (8%)	0.24
NSVT, n	11/63 (17%)	7/77 (9%)	0.14
Sustained ventricular tachycardia, n	-	-	-
Ventricular fibrillation/aborted sudden cardiac death	0/227 (0%)	1/214 (0%)	0.36
Atrial fibrillation	21/227 (9%)	15/214 (7%)	0.39
*CMR diagnosis*			
Normal heart	87/227 (38%)	113/214 (53%)	**0.002**
Ischemic heart disease	51/227 (22%)	32/214 (15%)	**0.04**
HNDCM/dilated cardiomyopathy	24/227 (11%)	12/214 (6%)	0.06
Myocarditis	19/227 (8%)	20/214 (9%)	0.72
Hypertensive heart disease	15/227 (7%)	6/214 (3%)	0.06
HCM and phenocopies	12/227 (5%)	7/214 (3%)	0.30
Takotsubo syndrome	2/227 (1%)	1/214 (1%)	0.60
Arrhythmogenic cardiomyopathy	3/227 (1%)	4/214 (2%)	0.65
Cardiac mass or tumor	2/227 (1%)	1/214 (0%)	0.60
Extracardiac mass	3/227 (1%)	5/214 (2%)	0.42
Congenital heart disease	1/227 (0%)	1/214 (0%)	0.97
Pericardial disease	8/227 (4%)	12/214 (6%)	0.29
*CMR characteristics*			
LV end diastolic volume, mL	151 (122-190)	142 (120-171)	**0.03**
LV mass, grams	112 (82-143)	98 (74-123)	**<0.001**
LV ejection fraction, %	59 (50-64)	61 (56-65)	**0.008**
Right ventricular end diastolic volume, mL	138 (113-163)	138 (113-171)	0.75
Right ventricular ejection fraction, %	60 (55-66)	60 (56-65)	0.63
Left atrial volume, mL	55 (41-70)	50 (40-67)	0.18
Right atrial volume, mL	46 (34-63)	47 (35-60)	0.67
T1-mapping, msec	1000 (982-1029)	998 (977-1018)	0.12
T2-mapping, msec	47 (45-49)	47 (45-48)	0.07
LGE presence, n	88/219 (40%)	67/206 (33%)	0.10
LGE pattern: subendocardial, n	33/88 (38%)	21/67 (31%)	0.43
LGE pattern: midwall, n	32/88 (37%)	30/67 (45%)	0.29
LGE pattern: subepicardial, n	13/88 (15%)	15/67 (23%)	0.21
LGE pattern: transmural, n	24/88 (27%)	11/67 (16%)	0.11
LGE: septal wall, n	40/88 (45%)	27/67 (41%)	0.57
LGE: anterior wall, n	28/88 (32%)	15/67 (23%)	0.21
LGE: lateral wall, n	34/88 (39%)	29/67 (43%)	0.56
LGE: inferior wall, n	38/88 (43%)	35/67 (53%)	0.23
LGE: apex, n	26/88 (30%)	16/67 (24%)	0.46
LGE: papillary muscles, n	6/88 (7%)	3/67 (5%)	0.55
LGE: right ventricle, n	2/88 (2%)	4/67 (6%)	0.23
LGE, number of segments (LGE+ patients)	2 (1-5)	2 (1-3)	0.33
Mitral annulus, systole, mm	27 ± 5	27 ± 5	0.82
Mitral annulus, diastole, mm	29 ± 5	29 ± 5	0.11
Mitral regurgitation ≥ mild	11/227 (5%)	6/214 (3%)	0.27
MVP, n	3/227 (1%)	26/214 (12%)	**<0.01**
MVP extent, mm	2.9 (2.1-2.9)	2.7 (2.0-4.2)	0.86
Bi-leaflet MVP, n	-	8/214 (4%)	**0.003**

*CMR* cardiac magnetic resonance, *ECG* electrocardiogram, *HCM* hypertrophic cardiomyopathy, *HNDCM* hypokinetic non-dilated cardiomyopathy, *LGE* late gadolinium enhancement, *LV* left ventricle, *MAD* mitral annulus disjunction, *MVP* mitral valve prolapse, *n* number of patients, *NSVT* non-sustained ventricular tachycardia, *PCI* percutaneous coronary intervention, *VEB* ventricular ectopic beat.

Bold numbers indicate statistically significant results.

### 3.2 MAD in the study population

Two hundred and fourteen out of four hundred and forty-one (49%) patients presented with MAD (Table 1). The most frequent MAD location was the LV inferior wall in the 2-chamber view (173/441 (39%) of patients; Table 2), followed by the anterior wall (104/441 (24%)), antero-lateral segment in the 4-chamber view (92/441 (21%)), and, lastly, infero-lateral segment in the 3-chamber view (87/441 (20%)). The prevalence of MAD greater than 4 mm and 6 mm was 14% (63/441) and 3% (15/441), respectively (Table 2).

**Table 2:** MAD prevalence in the study population according to MAD definition.

MAD cut-off		≥1 mm	≥2 mm	≥4 mm	≥6 mm
Patients with MAD, n (%)	All views	214/441 (49%)	173/441 (39%)	63/441 (14%)	15/441 (3%)
	3-chamber view	87/441 (20%)	64/441 (14%)	29/441 (7%)	7/441 (2%)
	2-chamber view, anterior wall	104/441 (24%)	70/441 (16%)	25/441 (6%)	7/441 (2%)
	2-chamber view, inferior wall	173/441 (39%)	127/441 (29%)	43/441 (10%)	8/441 (2%)
	4-chamber view	92/441 (21%)	60/441 (14%)	15/441 (3%)	5/441 (1%)

*MAD mitral annulus disjunction*.

Patients with MAD were younger (55 vs 57 years, p = 0.04) than patients without MAD with no differences in sex, cardiovascular risk factors, symptoms, and baseline ventricular arrhythmias. However, patients with MAD ≥4 mm had a greater prevalence of ≥10,000 VEBs/day compared to those with MAD less than 4 mm or no MAD (6/28 (21%) vs 2/112 (2%), p < 0.001) (Supplementary Table 1).

Patients without MAD were more frequently affected by ischemic heart disease (51/227 (22%) vs 32/214 (15%), p = 0.04) and presented with greater LV end diastolic volumes (151, 122-190 mL vs 142, 120-171 mL; p = 0.03), greater LV mass (112, 82-143 g vs 98, 74-123 g; p < 0.001), and lower LV ejection fraction (59, 50-64% vs 61, 56-65%; p = 0.008) than patients with MAD. There was no difference in pre-contrast T1-mapping, T2-mapping, LGE presence and extent, mitral annulus dimensions, and significant mitral regurgitation between patients with and without MAD (Table 1).

### 3.3 MAD in patients with and without MVP

The prevalence of MVP was higher in patients with MAD than in those without MAD (26/214 12% vs 3/227 1%; p < 0.01), and all 8 patients with bi-leaflet MVP presented with MAD (Table 1). Patients with MVP showed greater MAD prevalence (26/29 (90%) vs 188/412 (46%); p < 0.001; Table 3) and extent (4.2, 3.0-5.7 mm vs 2.8, 1.9-4.0 mm; p < 0.001) than those without MVP. Patients with MVP also showed a greater prevalence of MAD ≥4 mm (14/29 (48%) vs 49/412 (12%); p < 0.001) and MAD ≥6 mm (3/29 (10%) vs 12/412 (3%); p = 0.03) (Table 3).

**Table 3:** MAD characteristics in patients with and without MVP.

	Patients with MVP (n = 29)	Patients without MVP (n = 412)	p value
Age	53 (46-70)	56 (44-69)	0.68
Mitral annulus, systole, mm	31 ± 5	27 ± 5	**<0.001**
Mitral annulus, diastole, mm	29 ± 5	29 ± 5	0.34
*All views*			
MAD, n	26/29 (90%)	188/412 (46%)	**<0.001**
MAD ≥4 mm, n	14/29 (48%)	49/412 (12%)	**<0.001**
MAD ≥6 mm, n	3/29 (10%)	12/412 (3%)	**0.03**
MAD extent, mm (MAD+ patients)	4.2 (3.0-5.7)	2.8 (1.9-4.0)	**<0.001**
*3-chamber view*			
MAD, n	18/29 (62%)	63/412 (15%)	**<0.001**
MAD ≥4 mm, n	6/29 (21%)	18/412 (4%)	**<0.001**
MAD ≥6 mm, n	1/29 (4%)	4/412 (1%)	0.22
MAD extent, mm (MAD+ patients)	2.5 (0-4.0)	0 (0-1.8)	**<0.001**
*2-chamber view, anterior wall*			
MAD, n	14/29 (48%)	86/412 (21%)	**<0.001**
MAD ≥4 mm, n	3/29 (10%)	14/412 (3%)	0.06
MAD ≥6 mm, n	1/29 (4%)	4/412 (1%)	0.22
MAD extent, mm (MAD+ patients)	1.4 (0-3.0)	0 (0-2.1)	0.30
*2-chamber view, inferior wall*			
MAD, n	19/29 (66%)	144/412 (35%)	**<0.001**
MAD ≥4 mm, n	5/29 (17%)	26/412 (6%)	**0.026**
MAD ≥6 mm, n	1/29 (4%)	5/412 (1%)	0.32
MAD extent, mm (MAD+ patients)	2.6 (1.0-3.5)	2.0 (1.0-3.4)	0.39
*4-chamber view*			
MAD, n	19/29 (66%)	65/412 (16%)	**<0.001**
MAD ≥4 mm, n	5/29 (17%)	8/412 (2%)	**<0.001**
MAD ≥6 mm, n	2/29 (7%)	0/412	**<0.001**
MAD extent, mm (MAD+ patients)	2.1 (0-3.8)	0 (0-1.5)	**<0.001**

*MAD* mitral annulus disjunction, *MVP* mitral valve prolapse, *n* number of patients.

Bold numbers indicate statistically significant results.

### 3.4 Associates of MAD

The presence (odds ratio [OR] 9.7, 95% confidence interval [CI] 2.85-33; p < 0.001) and extent (OR 2.37; 95% CI 1.49-3.75; p < 0.001) of MVP were the only morpho-functional variables associated with the presence of MAD ≥1 mm at multivariable regression analysis (Table 4). Similar results were replicated for MAD ≥4 mm and ≥6 mm. Moreover, the baseline VEBs ≥10,000/day were associated with MAD ≥4 mm in a multivariable model considering MVP presence (OR 13.3; 95% CI 2.32-76.44; p = 0.004) or MVP extent (OR 14.64; 95% CI 2.6-82.25; p = 0.002) (Supplementary Tables 3 and 4).

**Table 4:** Univariable and multivariable logistic regression analysis of determinants associated with MAD presence in the study population.

Variables	Univariable analysis			Multivariable analysis (Model 1: MVP presence)			Multivariable analysis (Model 2: MVP extent)		
	OR	95% CI	p value	OR	95% CI	p value	OR	95% CI	p value
Age, years	**0.99**	**0.98; 1.00**	**0.04**	**0.99**	**0.97; 1.00**	**0.02**	**0.99**	**0.97; 1.00**	**0.02**
Female	1.19	0.81; 1.74	0.37						
Palpitations	1.13	0.57; 2.26	0.72						
VEBs ≥10,000 per day	2.58	0.50; 13.24	0.26						
NSVT	0.47	0.17; 1.30	0.15						
Atrial fibrillation	0.74	0.37; 1.48	0.39						
LV end diastolic volume, mL	**0.99**	**0.99; 1.00**	**0.004**	1.00	0.99; 1.00	0.12	1.00	0.99; 1.00	0.14
LV mass, grams	**0.99**	**0.99; 1.00**	**0.005**	1.00	0.99; 1.00	0.44	1.00	0.99; 1.00	0.48
LV ejection fraction, %	**1.03**	**1.01; 1.05**	**<0.001**	1.02	0.99; 1.04	0.19	1.02	0.99; 1.04	0.20
Left atrial volume, mL	0.99	0.99; 1.00	0.14						
T1-mapping, msec	1.00	0.99; 1.00	0.10						
T2-mapping, msec	0.95	0.89; 1.01	0.10						
LGE, presence	0.72	0.48; 1.07	0.10						
LGE: number of segments	**0.89**	**0.80; 0.99**	**0.03**	1.04	0.91; 1.19	0.59	1.04	0.90; 1.20	0.57
MVP, presence	**10.3**	**3.08; 34.65**	**<0.001**	**9.70**	**2.85; 33.00**	**<0.001**	-	-	-
MVP extent, mm	**2.45**	**1.55; 3.87**	**<0.001**	-	-	-	**2.37**	**1.49; 3.75**	**<0.001**
Mitral regurgitation ≥mild	0.57	0.21; 1.56	0.27						
Mitral annulus, systole, mm	1.00	0.97; 1.04	0.82						
Mitral annulus, diastole, mm	0.97	0.93; 1.00	0.11						
Ischemic heart disease	**0.61**	**0.37; 0.99**	**0.05**	0.80	0.42; 1.51	0.49	0.79	0.42; 1.50	0.47
HNDCM/dilated cardiomyopathy	0.50	0.24; 1.03	0.06						
Myocarditis	1.13	0.58; 2.18	0.72						
HCM and phenocopies	0.61	0.23; 1.57	0.30						
Arrhythmogenic cardiomyopathy	1.42	0.31; 6.43	0.65						
Congenital heart disease	1.06	0.07; 17.07	0.97						

*CI* confidence interval, *HCM* hypertrophic cardiomyopathy, *HNDCM* hypokinetic non-dilated cardiomyopathy, *LGE* late gadolinium enhancement, *LV* left ventricle, *MAD* mitral annulus disjunction, *MVP* mitral valve prolapse, *NSVT* non-sustained ventricular tachycardia, *OR* odds ratio, *VEB* ventricular ectopic beat.

Bold numbers indicate statistically significant results.

The presence (ß = 2.45; 95% CI 1.3-3.6; p < 0.001) and extent (ß = 0.78; 95% CI 0.43-1.13; p < 0.001) of MVP and VEBs ≥10,000/day (ß = 1.76; 95% CI 0.26-3.26; p = 0.02; ß = 2.01; 95% CI 0.52-3.5; p = 0.009) were the only variables associated with the extent of MAD at multivariable regression analysis (Table 5). The extent of MAD positively correlated with the extent of MVP (r = 0.48; p = 0.006) and mitral annulus measured in systole (r = 0.34; p < 0.001) (Supplementary Figs. 1 and 2).

**Table 5:** Univariable and multivariable linear regression analysis of determinants associated with MAD extent in patients with MAD.

Variables	Univariable analysis		Multivariable analysis (Model 1: MVP presence)		Multivariable analysis (Model 2: MVP extent)	
	p value	β (95% CI)	p value	β (95% CI)	p value	β (95% CI)
Age, years	**0.02**	**−0.012 (−0.23; 0.002)**	0.39	-0.009 (−0.315; 0.012)	0.48	-0.008 (−0.030; 0.014)
Female	0.79	0.05 (−0.33; 0.44)	-	-	-	-
Palpitations	0.90	0.04 (−0.66; 0.74)	-	-	-	-
VEBs ≥ 10,000 per day	**0.004**	**2.29 (0.72; 3.85)**	**0.02**	**1.76 (0.26; 3.26)**	**0.009**	**2.01 (0.52; 3.50)**
NSVT	0.38	−0.50 (−0.61; 0.61)	-	-	-	-
Ventricular fibrillation/aborted sudden cardiac death	0.46	1.65 (−2.78; 6.08)	-	-	-	-
Atrial fibrillation	0.88	−0.05 (−0.74; 0.64)	-	-	-	-
LV end diastolic volume, mL	**0.04**	**−0.004 (−0.007; 0)**	0.87	0.001 (−0.008; 0.009)	0.75	0.001 (−0.007; 0.009)
LV mass, grams	0.15	−0.003 (−0.006; 0.001)	-	-	-	-
LV ejection fraction, %	**0.005**	**0.02 (0.01; 0.04)**	0.86	−0.003 (−0.040; 0.034)	0.94	-0.001 (−0.038; 0.035)
Left atrial volume, mL	0.36	−0.004 (−0.012; 0.004)	-	-	-	-
T1-mapping, msec	0.51	−0.001 (−0.004; 0.002)	-	-	-	-
T2-mapping, msec	0.27	−0.002 (−0.006;0.002)	-	-	-	-
LGE, presence	0.41	−0.17 (−0.57; 0.23)	-	-	-	-
LGE: number of segments	0.08	−0.86 (−0.18; 0.01)	-	-	-	-
MVP, presence	**<0.001**	**2.52 (1.80; 3.25)**	**<0.001**	**2.45 (1.30; 3.60)**	**-**	**-**
MVP extent, mm	**<0.001**	**0.82 (0.60; 1.05)**	-	-	**<0.001**	**0.78 (0.43; 1.13)**
Mitral regurgitation ≥mild	0.60	−0.27 (−1.24; 0.72)	-	-	-	-
Mitral annulus, systole, mm	0.96	−0.001 (−0.040; 0.038)	**-**	**-**	**-**	**-**
Mitral annulus, diastole, mm	0.59	−0.01 (−0.05; 0.03)	-	-	-	-
Ischemic heart disease	**0.02**	**−0.59 (−1.07;−0.11)**	0.51	−0.38 (−1.51; 0.75)	0.50	−0.38 (−1.51; 0.74)
HNDCM/dilated cardiomyopathy	0.31	−0.35 (−1.04; 0.34)	-	-	-	-
Myocarditis	0.38	0.30 (−0.37; 0.96)	-	-	-	-
HCM and phenocopies	0.29	−0.50 (−1.43; 0.43)	-	-	-	-
Arrhythmogenic cardiomyopathy	0.14	1.14 (−0.37; 2.65)	-	-	-	-
Congenital heart disease	0.86	−0.26 (−3.08; 2.55)	-	-	-	-

*CI* confidence interval, *HCM* hypertrophic cardiomyopathy, *HNDCM* hypokinetic non-dilated cardiomyopathy, *LGE* late gadolinium enhancement, *LV* left ventricle, *MAD* mitral annulus disjunction, *MVP* mitral valve prolapse, *n* number of patients, *NSVT* non-sustained ventricular tachycardia, *VEB* ventricular ectopic beat.

Bold numbers indicate statistically significant results.

### 3.5 MAD reproducibility

Detection of MAD presence showed very good intra-observer (Cohen's kappa = 0.90) and good inter-observer (Cohen's kappa = 0.78) agreement for single patients (Supplementary Tables 5 and 6). Measurements of MAD extent showed excellent intra-observer (ICC = 0.93; bias −0.13, 95% limits of agreement: +1.12, −1.37 mm) and good inter-observer (ICC = 0.77; bias −0.25, 95% limits of agreement: +1.90, −2.40 mm) reliability (Supplementary Tables 5 and 6; Supplementary Fig. 3).

### 3.6 Echocardiography subgroup

Among the 16 patients with available TTE, the prevalence of MAD was higher at CMR (9/16, 56%) than at TTE (4/16, 25%), respectively. All patients showing MAD at TTE had MAD at CMR, too. The patients with MAD detected both at CMR and TTE had greater extent (6, 4.2-6.4 mm) than those showing MAD at CMR only (1.5, 1.2-2.5 mm, Supplementary Table 7).

### 3.7 Study endpoint

**Fig. 3:**
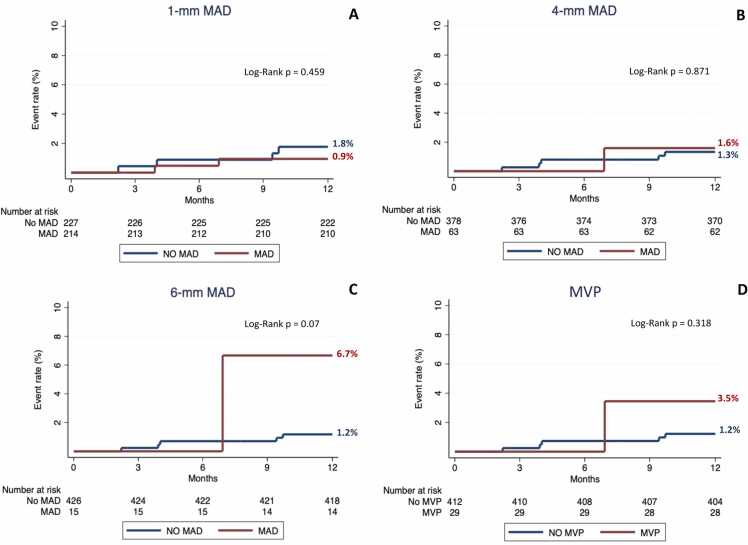
Kaplan-Meier curves for the presence or absence of MAD ≥1 mm (A), presence of MAD ≥4 mm (B), presence of MAD ≥6 mm (C), and presence or absence of MVP (D). *MAD* mitral annulus disjunction, *MVP* mitral valve prolapse.

During the 12-month follow-up, 6/441 patients (1.4%) experienced the composite endpoint. Five patients experienced unexplained syncope, and one patient sustained ventricular tachycardia. The presence of MAD ≥1 mm (0.9% vs 1.8%; p = 0.46), MAD ≥4 mm (1.6% vs 1.3%; p = 0.87), or MVP (3.5 vs 1.2%; p = 0.32) were not significantly associated with the study endpoint. Patients with MAD ≥6 mm showed a trend toward a higher likelihood of the study endpoint than patients without, however with borderline significance (6.7% vs 1.2%; p = 0.07; Fig. 3). Firth and Poisson univariate regression analyses for the composite endpoint, however, were non-significant (Supplementary Table 8).

## 4 Discussion

There is increasing interest in MAD, which represents an imaging marker with unknown clinical significance but is proposed as a potential risk marker of SCD [2], [3], [4], [5], [6], [9], [10], [11]. Most studies focused on selected patients with MVP or a high burden of ventricular arrhythmias. We characterized MAD and explored the association between this structural abnormality with other CMR features and ventricular arrhythmias in a cohort of consecutive patients undergoing clinically indicated CMR. The main study findings are i) a MAD of limited severity (i.e., less than 4 mm) is a common and benign condition in the clinical arena; ii) severe MAD is uncommon and its arrhythmogenic potentials need further prospective studies to be completely elucidated.

### 4.1 MAD of limited entity as a common and benign finding

The prevalence of MAD ≥1 mm was around 50% in the whole cohort of patients, approaching 90% in patients with MVP. Notably, this abnormality was not associated with morpho-functional or tissue alterations at CMR, symptoms or ventricular arrhythmias at baseline or follow-up. In contrast to our findings, Dejgaard et al. in a cross-sectional study of 112 patients with MAD (defined as ≥1 mm; median value of MAD extent: 3.0 mm) found a high prevalence of ventricular arrhythmias (34%) and malignant/severe ventricular arrhythmias (12%); notably, arrhythmic events were not associated with MVP but with a larger longitudinal extent of MAD in the postero-lateral wall, among others [11]. Differences in patient selection can explain these discordant results. Our cohort showed a massively lower prevalence of malignant ventricular arrhythmias (1% vs 12%) and MVP (7% vs 78%), highlighting better generalizability of our study findings. Importantly, the present study aligns with recent findings downscaling the potentially malignant role of an isolated MAD of limited entity. In a CMR-based multicenter study including 474 patients with MVP without comorbidities, significant mitral regurgitation, or LV dysfunction, the presence of MAD was not longitudinally associated with a composite endpoint, including sustained ventricular tachycardia, (aborted) SCD, or unexplained syncope [4]. The authors evaluated MAD only in 3-chamber view and they found it in 68% of patients. Interestingly, by replicating this approach to the subgroup of patients with MVP, we would have found a comparable MAD prevalence (i.e., 62%). Recently, Zugwitz et al. investigated MAD in a large-scale population of volunteers undergoing non-contrast CMR [18]. By using a cut-off of 1 mm, the authors confirmed a very high prevalence of MAD (i.e., 76%). In line with our findings, the authors found the highest and lowest prevalences of MAD in the 2-chamber and 3-chamber view, respectively. MAD of limited severity at the level of P1 and P3 scallops explored by the 2-chamber view might reflect a common and benign extension of fibrous tissue from the fibrous trigones to the mitral annulus, which generally spares the P2 scallop, which is explored by the 3-chamber view [22].

### 4.2 Association between MAD and MVP

The present study concurs to highlight the strict link between MAD and MVP. The presence and extent of MVP were the only morpho-functional abnormalities significantly associated with the presence and extent of MAD. Notably, patients with MVP showed a higher MAD prevalence in all long-axis views, indicating a more extensive disjunction along the entire mitral annulus. At autopsy, Hutchins et al. [1] were the first to unveil this robust association by showing MAD in 92% of hearts with MVP, perfectly matching our results, and only in 5% of hearts without MVP. The authors postulated that MAD was the anatomical substrate leading to MVP by entailing repeated traction on the mitral leaflets [1], [5].

### 4.3 Extended MADs as potential arrhythmogenic entities

It is conceivable that MAD of greater severity parallel higher degrees of stretch on the posterior myocardial wall and papillary muscles of the LV, mechanically inducing ventricular arrhythmias [1], [6]. Accordingly, we found that a high burden of VEBs predicted a MAD ≥4 mm, whereas a MAD ≥6 mm was associated with a numerically higher occurrence of the study endpoint with borderline statistical significance. In line with this, MAD ≥8.5 mm predicted non-sustained ventricular tachycardia in a population of patients with MVP [23], and SCD only occurred in the presence of MAD >10 mm in a population with Marfan disease [24]. Significant traction exerted by greater MAD on the LV, which might be unveiled by strain imaging [25], [26], can also induce the development of myocardial fibrosis [27], which has been robustly associated with malignant ventricular arrhythmias in patients with MVP [4], [28]. A greater amount of myocardial fibrosis has indeed been described in patients with MVP and extended MAD [2]. Local stretch and fibrosis might induce QT prolongation and exert ectopic foci from Purkinje fibers, which extend into the papillary muscles [29]. In this way, mechanical triggers related to extended MAD might translate into electrical instability. Thus, our study findings and previous literature suggest that especially pronounced MADs might play an arrhythmogenic role [1], [6], which needs further clarification. The heterogeneity of our study population, including several confounders for myocardial fibrosis or strain alterations, precludes us from deepening the relationship between MAD extent and tissue and functional alterations. Dedicated studies might clarify whether deformation imaging alterations [25] and increased values of native T1-mapping or extracellular volume [30] or non-ischemic LGE [4], [28] might be complementary with extended MADs in capturing patients at increased risk of ventricular arrhythmias.

### 4.4 Methodological issues in MAD assessment

The higher the disjunction threshold, the lower the MAD prevalence. In our study population, MAD prevalence would have been 3.5 times and 16 times decreased changing the disjunction reference from 1 mm to 4 mm and 6 mm, respectively. Several studies have settled reference ≤1 mm [2], [3], [11], [18] for MAD detection, and this was the threshold used in our study to explore the prevalence of MAD along the whole spectrum of severity.

Given that MAD is a circumferential phenomenon [11], our data confirm that its prevalence increases with the number of long-axis views analyzed.

Konda et al. searched MAD through echocardiography in 1439 consecutive patients, documenting it in 125 cases (9%), of which only 15 (12% of MAD patients) showed MVP [31]. The absolute excess of MAD+/MVP− patients over MAD+/MVP+ patients aligns with our results in supporting that MAD is a common condition even in patients without MVP. Echocardiography has shown lower reproducibility and accuracy than CMR in assessing MAD because of a dependency on acoustic windows and lower image resolution [32]. This issue is confirmed by our data in which approximately half of the MADs were not evident at echocardiography, especially those of limited extent. By confirming optimal CMR intra-operator and inter-operator agreement for MAD detection and measurement, our findings also confirm CMR as an ideal imaging tool to assess MAD [12]. Overall, our results show that discrepancies in the imaging modality, the MAD threshold, and location dramatically impact the prevalence of this condition in a study cohort. A consensus statement of experts would help standardize MAD assessment, improving the comparability of study results from different study groups.

### 4.5 Study limitations

Several limitations have to be acknowledged. First, the single-center design represents a weakness, although it allows high reproducibility and robustness in MAD measurements and CMR analysis. Second, the rather low number of events and the relatively short follow-up time might have increased the risk of type II error; however, in a cohort of unselected patients undergoing CMR, the majority of which are “normal,” a very low incidence of the composite endpoint was expected. To tackle this, additional sensitivity analyses were employed; however, the survival analysis is to be considered exploratory, and further prospective, well-powered studies are needed. The lack of a planned, systematic ECG-Holter monitoring after CMR reflects the retrospective design and might lead to underestimation of ventricular arrhythmias at follow-up. However, the predefined study endpoint included clinically relevant, malignant ventricular arrhythmias unlikely to elude clinical follow-up. Third, mitral leaflet thickness was not assessed because this is below the spatial resolution of CMR and subjected to partial volume averaging [33]. Fourth, the heterogeneity of the study population inevitably impacts morpho-functional parameters and tissue characterization, precluding any inferences with MAD characteristics. Fifth, an analysis of myocardial strain that could unveil subclinical changes owing to MAD was not performed because of the heterogeneity of the study cohort included. Future investigations with different cohorts of patients will be necessary to explore the potential interplay between MAD and cardiac structure, function, and tissue properties. Sixth, TTE was available only in a small minority of patients, and dedicated studies remain needed to evaluate the role of imaging modalities in MAD characterization of unselected cohorts of patients. Finally, we focused on selected patients undergoing clinically referred CMR in a tertiary center and the findings of the present study cannot be extrapolated to the general population. However, most patients were asymptomatic for palpitations and syncope and did not present with MVP. On the contrary, most patients presented a structurally normal heart or with ischemic heart disease. Thus, we believe that the key messages of the study remain consistent.

## 5 Conclusions

MAD of limited entity was a common and benign finding in consecutive patients clinically referred to CMR. Greater extents of MAD (i.e., ≥4-6 mm) were rarer and showed association with ventricular arrhythmias at baseline. MVP was the only morpho-functional abnormality associated with the presence and extent of MAD. The mid-term prognosis of MAD seems overall benign, but the numerically higher occurrence of adverse events at follow-up in patients with extended MAD needs further clarification. Prospective, well-powered studies on larger cohorts with longer follow-up times are warranted to search for potential “malignant MAD extents” to improve patients’ risk stratification.

## Funding

None.

## Author contributions

**Francesco Cannata:** Writing – review and editing, Methodology, Investigation, Data curation. **Fabio Fazzari:** Writing – review and editing, Methodology, Investigation, Data curation. **Marzia Olivieri:** Writing – review and editing, Methodology, Investigation, Data curation. **Renato Maria Bragato:** Writing – review and editing, Visualization, Validation, Supervision. **Stefano Figliozzi:** Writing – review and editing, Writing – original draft, Visualization, Validation, Supervision, Methodology, Investigation, Formal analysis, Data curation, Conceptualization. **Georgios Georgiopoulos:** Writing – review and editing, Visualization, Validation, Supervision. **Kamil Stankowski:** Writing – review and editing, Writing – original draft, Methodology, Investigation, Formal analysis, Data curation. **Pier-Giorgio Masci:** Writing – review and editing, Visualization, Validation, Supervision. **Lara Tondi:** Writing – review and editing, Formal analysis, Data curation. **Lorenzo Monti:** Writing – review and editing, Visualization, Validation, Supervision, Data curation. **Federica Catapano:** Writing – review and editing, Formal analysis, Data curation. **Gianluigi Condorelli:** Writing – review and editing, Visualization, Validation, Supervision. **Mauro Gitto:** Writing – review and editing, Investigation, Formal analysis. **Marco Francone:** Writing – review and editing, Writing – original draft, Visualization, Validation, Supervision, Project administration, Methodology, Investigation, Formal analysis, Data curation, Conceptualization. **Costanza Lisi:** Writing – review and editing, Methodology, Investigation, Data curation. **Sara Bombace:** Writing – review and editing, Methodology, Investigation, Data curation.

## Ethics approval and consent

The study protocol was performed according to the principles of the Declaration of Helsinki and was approved by the Ethics Committee of the institutional review board. All patients gave written consent to have their anonymized clinical data used for scientific purposes.

## Consent for publication

Not applicable.

## Declaration of competing interests

The authors declare that they have no known competing financial interests or personal relationships that could have appeared to influence the work reported in this paper.

## Data Availability

The data underlying this article will be shared on reasonable request to the corresponding author.
