# Supplementary material for: Mitral annulus disjunction in consecutive patients undergoing cardiovascular magnetic resonance: Where is the boundary between normality and disease?
Source: J Cardiovasc Magn Reson. 2024 Jul 4;26(2):101056. doi: 10.1016/j.jocmr.2024.101056 (PMC11334631; doi:10.1016/j.jocmr.2024.101056)
Supplement: Supplementary file 1 — Supplementary material. [file mmc1.docx]

**Online Supplement**

**I. Supplementary Methods**

**II. Supplementary Tables**

**III. Supplementary Figures**

**I. Supplementary Methods**

*CMR acquisition protocol*

Cine images were acquired using breath-hold steady-state free-precession sequences. Patient-specific shimming was performed, and a shim box was placed tightly over the heart as recommended. T1 and T2 measurement was performed using a Modified Looked Locker Inversion recovery sequence (MOLLI) before contrast. MOLLI sequences were acquired in mid-ventricular short axis. Optimal gating and breath-holding were ensured, and raw images and R2 maps were examined for potential image artifacts during scanning, to allow an immediate repeat of suboptimal measurement. Inline generated T1 and T2 maps were analyzed using Circle CVI42 station-version-5.13.7 (Circle Cardiovascular Imaging Inc. Calgary, Canada). T1 and T2 values were measured through a region of interest in the mid-septal wall, taking care to avoid the blood-myocardial boundary. Late Gadolinium Enhancement (LGE) images were acquired >8 minutes after an i.v. bolus of gadolinium-based contrast-agent (Gadovist; Bayer Schering Pharma, Berlin, Germany) by phase-sensitive-inversion-recovery gradient-echo sequence. Inversion-time was individually optimized to null normal myocardium based on Look-Locker images.

**II. Supplementary Tables**

**Supplementary Table 1. Clinical and CMR characteristics of the study population according to presence of MAD (≥4 mm).**

| **Variables** | **MAD absence**  **(n=378)** | **MAD presence**  **(n=63)** | **p-value** |
| --- | --- | --- | --- |
| ***Demographic and clinical data*** |  |  |  |
| Age, years | 56 (45-69) | 51 (37-66) | **0.05** |
| Female, n | 147/378 (39%) | 27/63 (43%) | 0.55 |
| Athletes, n | 17/378 (5%) | 1/63 (2%) | 0.28 |
| Previous PCI, n | 79/378 (21%) | 6/63 (10%) | 0.06 |
| Hypertension, n | 147/378 (39%) | 15/63 (24%) | **0.03** |
| Diabetes, n | 42/378 (11%) | 4/63 (6%) | 0.32 |
| Hypercholesterolemia, n | 129/378 (34%) | 16/63 (25%) | 0.21 |
| No symptoms, n | 218/378 (58%) | 39/63 (62%) | 0.53 |
| Typical chest pain, n | 16/378 (4%) | 6/63 (10%) | 0.15 |
| Atypical chest pain, n | 36/378 (10%) | 4/63 (6%) | 0.42 |
| Palpitations, n | 32/378 (9%) | 3/63 (5%) | 0.31 |
| Dyspnoea, n | 77/378 (20%) | 11/63 (18%) | 0.59 |
| Unexplained syncope, n | 5/378 (1%) | 0/63 | 0.36 |
| 24-hour ECG Holter, n | 112/378 (30%) | 28/63 (44%) | **0.02** |
| VEBs **≥**10.000 per day, n | 2/112 (2%) | 6/28 (21%) | **<0.001** |
| NSVT, n | 15/112 (13%) | 3/28 (11%) | 0.70 |
| Sustained ventricular tachycardia, n | - | - | - |
| Ventricular fibrillation/aborted-sudden-cardiac-death | 1/378 (1%) | 0/63 | 0.62 |
| Atrial fibrillation | 30/378 (8%) | 6/63 (10%) | 0.67 |
| ***CMR analysis*** |  |  |  |
| Normal heart | 168/378 (43%) | 32/63 (51%) | 0.31 |
| Ischemic heart disease | 79/378 (21%) | 4/63 (6%) | **0.006** |
| HNDCM/dilated cardiomyopathy | 29/378 (8%) | 7/63 (11%) | 0.36 |
| Myocarditis | 31/378 (8%) | 8/63 (13%) | 0.24 |
| Hypertensive heart disease | 19/378 (5%) | 2/63 (3%) | 0.52 |
| HCM and phenocopies | 17/378 (5%) | 2/63 (3%) | 0.52 |
| Takotsubo syndrome | 3/378 (1%) | 0/63 | 0.48 |
| Arrhythmogenic cardiomyopathy | 5/378 (1%) | 2/63 (3%) | 0.63 |
| Cardiac mass or tumour | 3/378 (1%) | 0/63 | 0.48 |
| Extracardiac mass | 7/378 (2%) | 1/63 (2%) | 0.88 |
| Congenital heart disease | 2/378 (1%) | 0/63 | 0.56 |
| Pericardial disease | 15/378 (4%) | 5/63 (8%) | 0.16 |
| LV-end-diastolic-volume, ml | 145 (119-184) | 148 (124-161) | 0.74 |
| LV-mass, grams | 106 (77-135) | 94 (78-115) | **0.05** |
| LV-ejection-fraction, % | 60 (54-65) | 61 (56-65) | 0.4 |
| Right-ventricular-end-diastolic-volume, ml | 137 (113-165) | 152 (112-172) | 0.23 |
| Right-ventricular-ejection-fraction, % | 60 (55-66) | 59 (55-64) | 0.22 |
| Left atrial volume, ml | 53 (40-69) | 51 (40-65) | 0.88 |
| Right atrial volume, ml | 46 (34-62) | 49 (35-60) | 0.83 |
| T1-mapping, msec | 999 (978-1023) | 1001 (988-1020) | 0.95 |
| T2-mapping, msec | 47 (45-48) | 47 (45-48) | 0.93 |
| LGE presence, n | 133/363 (36%) | 22/62 (35%) | 0.86 |
| LGE-pattern:  subendocardial, n | 50/133 (38%) | 4/22 (18%) | 0.07 |
| LGE-pattern: midwall, n | 51/133 (38%) | 11/22 (50%) | 0.30 |
| LGE-pattern:  subepicardial, n | 21/133 (16%) | 7/22 (32%) | 0.07 |
| LGE-pattern:  transmural, n | 30/133 (23%) | 5/22 (23%) | 0.99 |
| LGE:septal-wall, n | 57/133 (43%) | 10/22 (46%) | 0.84 |
| LGE:anterior-wall, n | 39/133 (30%) | 4/22 (18%) | 0.27 |
| LGE:lateral-wall, n | 52/133 (39%) | 11/22 (50%) | 0.33 |
| LGE:inferior-wall, n | 58/133 (44%) | 15/22 (68%) | **0.04** |
| LGE:apex, n | 36/133 (27%) | 6/22 (27%) | 1.00 |
| LGE:papillary-muscles, n | 9/133 (7%) | 0/22 | 0.21 |
| LGE:right-ventricle, n | 5/133 (4%) | 1/22 (5%) | 0.87 |
| LGE, number of segments  (LGE+ patients) | 2 (1-4) | 2 (1-3) | 0.55 |
| Mitral annulus, systole, mm | 27 ± 5 | 27 ± 5 | 0.85 |
| Mitral annulus, diastole, mm | 29 ± 5 | 29 ± 4 | 0.51 |
| Mitral regurgitation ≥ mild | 15/378 (4%) | 2/63 (3%) | 0.76 |
| MVP, n | 15/378 (4%) | 14/63 (22%) | **<0.001** |
| MVP extent, mm | 2.5 (2.0-2.9) | 3.5 (2.7-4.2) | **0.02** |
| Bi-leaflet MVP, n | 1/378 (0%) | 7/63 (11%) | **<0.001** |

CMR: Cardiac Magnetic Resonance; HCM: Hypertrophic cardiomyopathy; HNDCM: hypokinetic non-dilated cardiomyopathy; LGE: Late-Gadolinium-Enhancement; LV: Left-Ventricle; MAD: Mitral-Annulus-Disjunction; MVP: Mitral-Valve-Prolapse; n: number of patients; NSVT: non-sustained-ventricular-tachycardia; PCI: Percutaneous-Coronary-Intervention; VEB: Ventricular-Ectopic-Beat.

**Supplementary Table 2. Clinical and CMR characteristics of the study population according to presence of MAD (≥6 mm).**

| **Variables** | **MAD absence**  **(n=426)** | **MAD presence**  **(n=15)** | **p-value** |
| --- | --- | --- | --- |
| ***Demographic and clinical data*** |  |  |  |
| Age, years | 56 (45-69) | 52 (28-28) | 0.36 |
| Female, n | 170/426 (40%) | 4/15 (27%) | 0.30 |
| Athletes, n | 18/426 (4%) | 0/15 | 0.42 |
| Previous PCI, n | 85/426 (20%) | 2/15 (13%) | 0.54 |
| Hypertension, n | 162/426 (38%) | 2/15 (13%) | **0.05** |
| Diabetes, n | 47/426 (11%) | 0/15 | 0.17 |
| Hypercholesterolemia, n | 153/390 (36%) | 3/15 (20%) | 0.27 |
| No symptoms, n | 247/426 (58%) | 10/15 (67%) | 0.50 |
| Typical chest pain, n | 21/426 (5%) | 1/15 (7%) | 0.92 |
| Atypical chest pain, n | 39/426 (9%) | 1/15 (7%) | 0.74 |
| Palpitations, n | 34/426 (8%) | 1/15 (7%) | 0.85 |
| Dyspnoea, n | 86/426 (20%) | 2/15 (13%) | 0.51 |
| Unexplained syncope, n | 5/426 (1%) | 0/15 | 0.67 |
| 24-hour ECG Holter, n | 134/426 (31%) | 6/15 (40%) | 0.48 |
| VEBs **≥**10.000 per day, n | 7/134 (5%) | 1/6 (17%) | 0.24 |
| NSVT, n | 17/134 (13%) | 1/6 (17%) | 0.46 |
| Sustained ventricular tachycardia, n | - | - | - |
| Ventricular fibrillation/aborted-sudden-cardiac-death | 1/426 (1%) | 0/15 | 0.83 |
| Atrial fibrillation | 34/426 (8%) | 2/15 (13%) | 0.39 |
| ***CMR analysis*** |  |  |  |
| Normal heart | 192/426 (45%) | 8/15 (53%) | 0.53 |
| Ischemic heart disease | 82/426 (19%) | 1/15 (7%) | 0.22 |
| HNDCM/dilated cardiomyopathy | 35/426 (8%) | 1/15 (7%) | 0.83 |
| Myocarditis | 37/426 (9%) | 2/15 (13%) | 0.53 |
| Hypertensive heart disease | 21/426 (5%) | 0/15 | 0.38 |
| HCM and phenocopies | 19/426 (5%) | 0/15 | 0.40 |
| Takotsubo syndrome | 3/426 (1%) | 0/15 | 0.74 |
| Arrhythmogenic cardiomyopathy | 6/426 (1%) | 1/15 (7%) | 0.11 |
| Cardiac mass or tumour | 3/426 (1%) | 0/15 | 0.74 |
| Extracardiac mass | 8/426 (2%) | 0/15 | 0.59 |
| Congenital heart disease | 2/426 (1%) | 0/15 | 0.79 |
| Pericardial disease | 18/426 (4%) | 2/15 (13%) | 0.10 |
| LV-end-diastolic-volume, ml | 145 (120-182) | 156 (131-161) | 0.66 |
| LV-mass, grams | 104 (77-133) | 113 (89-115) | 0.89 |
| LV-ejection-fraction, % | 60 (54-65) | 61 (56-66) | 0.56 |
| Right-ventricular-end-diastolic-volume, ml | 137 (113-166) | 169 (152-180) | **0.02** |
| Right-ventricular-ejection-fraction, % | 60 (55-66) | 58 (52-63) | 0.08 |
| Left atrial volume, ml | 53 (40-69) | 51 (40-79) | 0.79 |
| Right atrial volume, ml | 46 (34-61) | 49 (40-75) | 0.30 |
| T1-mapping, msec | 1000 (979-1022) | 998 (985-1005) | 0.49 |
| T2-mapping, msec | 47 (45-48) | 47 (46-48) | 0.49 |
| LGE presence, n | 148/410 (36%) | 7/15 (47%) | 0.40 |
| LGE-pattern:  subendocardial, n | 53/148 (36%) | 1/7 (14%) | 0.24 |
| LGE-pattern: midwall, n | 58/148 (39%) | 4/7 (57%) | 0.34 |
| LGE-pattern:  subepicardial, n | 27/148 (18%) | 1/7 (14%) | 0.78 |
| LGE-pattern:  transmural, n | 34/148 (23%) | 1/7 (14%) | 0.59 |
| LGE:septal-wall, n | 64/148 (44%) | 3/7 (43%) | 0.97 |
| LGE:anterior-wall, n | 42/148 (29%) | 1/7 (14%) | 0.41 |
| LGE:lateral-wall, n | 61/148 (41%) | 2/7 (29%) | 0.51 |
| LGE:inferior-wall, n | 70/148 (48%) | 3/7 (43%) | 0.81 |
| LGE:apex, n | 41/148 (28%) | 1/7 (14%) | 0.43 |
| LGE:papillary-muscles, n | 9/148 (6%) | 0/7 | 0.50 |
| LGE:right-ventricle, n | 5/148 (3%) | 1/7 (14%) | 0.15 |
| LGE, number of segments  (LGE+ patients) | 2 (1-4) | 2 (1-2) | 0.17 |
| Mitral annulus, systole, mm | 27 ± 5 | 28 ± 6 | 0.26 |
| Mitral annulus, diastole, mm | 29 ± 5 | 30 ± 5 | 0.27 |
| Mitral regurgitation ≥ mild | 16/426 (4%) | 1/15 (7%) | 0.56 |
| MVP, n | 26/426 (6%) | 3/15 (20%) | **0.03** |
| MVP extent, mm | 2.6 (2.0-4.0) | 4.0 (2.7-4.4) | 0.21 |
| Bi-leaflet MVP, n | 6/426 (1%) | 2/15 (13%) | **<0.001** |

CMR: Cardiac Magnetic Resonance; HCM: Hypertrophic cardiomyopathy; HNDCM: hypokinetic non-dilated cardiomyopathy; LGE: Late-Gadolinium-Enhancement; LV: Left-Ventricle; MAD: Mitral-Annulus-Disjunction; MVP: Mitral-Valve-Prolapse; n: number of patients; NSVT: non-sustained-ventricular-tachycardia; PCI: Percutaneous-Coronary-Intervention; VEB: Ventricular-Ectopic-Beat.

**Supplemental Table 3. Univariable and multivariable logistic regression analysis of determinants associated with MAD ≥4 mm in the study population.**

| **Variables** | **Univariable analysis** | | | **Multivariable analysis**  **(Model 1: MVP presence)** | | | **Multivariable analysis**  **(Model 2: MVP extent)** | | |
| --- | --- | --- | --- | --- | --- | --- | --- | --- | --- |
|  | **OR** | **95% CI** | **P Value** | **OR** | **95% CI** | **P Value** | **OR** | **95% CI** | **P Value** |
| Age, years | **0.98** | **0.97; 1.00** | **0.04** | 1.00 | 0.97; 1.03 | 0.11 | 1.00 | 0.98; 1.03 | 0.86 |
| Female | 1.18 | 0.69; 2.03 | 0.55 |  |  |  |  |  |  |
| Palpitations | 0.54 | 0.16; 1.82 | 0.32 |  |  |  |  |  |  |
| VEBs **≥**10.000 per day | **15.00** | **2.84; 79.24** | **0.001** | **13.30** | **2.32; 76.44** | **0.004** | **14.64** | **2.60; 82.25** | **0.002** |
| NSVT | 0.78 | 0.21; 2.89 | 0.70 |  |  |  |  |  |  |
| Atrial Fibrillation | 1.22 | 0.49; 3.06 | 0.67 |  |  |  |  |  |  |
| LV-end-diastolic-volume, ml | 1.00 | 0.99; 1.00 | 0.36 |  |  |  |  |  |  |
| LV-mass, grams | 1.00 | 0.99; 1.00 | 0.83 |  |  |  |  |  |  |
| LV-ejection-fraction, % | 1.02 | 0.99; 1.05 | 0.13 |  |  |  |  |  |  |
| Left atrial volume, ml | 1.00 | 0.99; 1.00 | 0.70 |  |  |  |  |  |  |
| T1-mapping, msec | 1.00 | 1.00; 1.00 | 0.72 |  |  |  |  |  |  |
| T2-mapping, msec | 1.00 | 0.98; 1.01 | 0.67 |  |  |  |  |  |  |
| LGE, presence | 0.95 | 0.52; 1.67 | 0.86 |  |  |  |  |  |  |
| LGE:number-of-segments | 0.94 | 0.81; 1.09 | 0.43 |  |  |  |  |  |  |
| MVP, presence | **6.91** | **3.15; 15.19** | **<0.001** | **5.25** | **1.59; 17.32** | **0.007** | ~~-~~ | ~~-~~ | ~~-~~ |
| MVP extent, mm | **1.96** | **1.51; 2.54** | **<0.001** | ~~-~~ | ~~-~~ | ~~-~~ | **1.67** | **1.16; 2.41** | **0.006** |
| Mitral regurgitation ≥mild | 0.79 | 0.18; 3.56 | 0.76 |  |  |  |  |  |  |
| Mitral annulus, systole, mm | 0.99 | 0.94; 1.05 | 0.85 |  |  |  |  |  |  |
| Mitral annulus, diastole, mm | 1.02 | 0.96; 1.08 | 0.51 |  |  |  |  |  |  |
| Ischemic heart disease | **0.26** | **0.09; 0.73** | **0.01** | 0.33 | 0.04; 2.85 | 0.32 | 0.33 | 0.04; 2.82 | 0.31 |
| HNDCM/dilated cardiomyopathy | 1.50 | 0.63; 3.60 | 0.36 |  |  |  |  |  |  |
| Myocarditis | 1.63 | 0.71; 3.73 | 0.25 |  |  |  |  |  |  |
| HCM and phenocopies | 0.70 | 0.16; 3.09 | 0.63 |  |  |  |  |  |  |
| Arrhythmogenic cardiomyopathy | 2.45 | 0.46; 12.89 | 0.29 |  |  |  |  |  |  |

CMR: Cardiac Magnetic Resonance; HCM: Hypertrophic cardiomyopathy; HNDCM: hypokinetic non-dilated cardiomyopathy; LGE: Late-Gadolinium-Enhancement; LV: Left-Ventricle; MAD: Mitral-Annulus-Disjunction; MVP: Mitral-Valve-Prolapse; n: number of patients; NSVT: non-sustained-ventricular-tachycardia; PCI: Percutaneous-Coronary-Intervention; VEB: Ventricular-Ectopic-Beat.

**Supplemental Table 4. Univariable and multivariable logistic regression analysis of determinants associated with MAD ≥6 mm in the study population.**

| **Variables** | **Univariable analysis** | | | **Multivariable analysis**  **(Model 1: MVP presence)** | | | **Multivariable analysis**  **(Model 2: MVP extent)** | | |
| --- | --- | --- | --- | --- | --- | --- | --- | --- | --- |
|  | **OR** | **95% CI** | **P Value** | **OR** | **95% CI** | **P Value** | **OR** | **95% CI** | **P Value** |
| Age, years | 0.99 | 0.96; 1.01 | 0.29 |  |  |  |  |  |  |
| Female | 0.54 | 0.17; 1.75 | 0.31 |  |  |  |  |  |  |
| Palpitations | 0.82 | 0.11; 6.45 | 0.85 |  |  |  |  |  |  |
| VEBs **≥**10.000 per day | 3.63 | 0.37; 35.40 | 0.27 |  |  |  |  |  |  |
| NSVT | 1.38 | 0.15; 12.50 | 0.78 |  |  |  |  |  |  |
| Atrial Fibrillation | 1.77 | 0.38; 8.19 | 0.73 |  |  |  |  |  |  |
| LV-end-diastolic-volume, ml | 1.00 | 0.99; 1.01 | 0.77 |  |  |  |  |  |  |
| LV-mass, grams | 1.00 | 0.98; 1.01 | 0.57 |  |  |  |  |  |  |
| LV-ejection-fraction, % | 1.02 | 0.97; 1.08 | 0.40 |  |  |  |  |  |  |
| Left atrial volume, ml | 1.00 | 0.98; 1.02 | 0.98 |  |  |  |  |  |  |
| T1-mapping, msec | 1.00 | 0.99; 1.00 | 0.66 |  |  |  |  |  |  |
| T2-mapping, msec | 0.99 | 0.94;1.05 | 0.85 |  |  |  |  |  |  |
| LGE, presence | 1.55 | 0.55; 4.36 | 0.41 |  |  |  |  |  |  |
| LGE:number-of-segments | 0.91 | 0.67; 1.25 | 0.57 |  |  |  |  |  |  |
| MVP, presence | **3.85** | **1.02; 14.48** | **0.05** | **9.70** | **2.85; 33.00** | **<0.0001** | ~~-~~ | ~~-~~ | ~~-~~ |
| MVP extent, mm | **1.57** | **1.08; 2.29** | **0.02** | ~~-~~ | ~~-~~ | ~~-~~ | **2.37** | **1.49; 3.75** | **<0.0001** |
| Mitral regurgitation ≥mild | 1.83 | 0.23; 14.79 | 0.57 |  |  |  |  |  |  |
| Mitral annulus, systole, mm | 1.06 | 0.96; 1.18 | 0.26 |  |  |  |  |  |  |
| Mitral annulus, diastole, mm | 1.06 | 0.95; 1.18 | 0.27 |  |  |  |  |  |  |
| Ischemic heart disease | 0.30 | 0.39; 2.31 | 0.25 |  |  |  |  |  |  |
| HNDCM/dilated cardiomyopathy | 0.80 | 0.10; 6.25 | 0.83 |  |  |  |  |  |  |
| Myocarditis | 1.62 | 0.35; 7.44 | 0.54 |  |  |  |  |  |  |
| Arrhythmogenic cardiomyopathy | 5.00 | 0.56; 44.36 | 0.15 |  |  |  |  |  |  |

CMR: Cardiac Magnetic Resonance; HNDCM: hypokinetic non-dilated cardiomyopathy; LGE: Late-Gadolinium-Enhancement; LV: Left-Ventricle; MAD: Mitral-Annulus-Disjunction; MVP: Mitral-Valve-Prolapse; n: number of patients; NSVT: non-sustained-ventricular-tachycardia; PCI: Percutaneous-Coronary-Intervention; VEB: Ventricular-Ectopic-Beat.

**Supplementary Table 5. Intra-observer agreement on MAD presence and MAD extent in the same view per single patient.**

|  | **Pearson** | **Cohen’s K Coefficient** | **Bias** | **Upper LOA** | **Lower LOA** | **ICC** |
| --- | --- | --- | --- | --- | --- | --- |
| ***All views*** | | | | | | |
| MAD, presence | 0.90 | 0.90 | - | - | - | - |
| MAD, mm | 0.88 |  | -0.13 | 1.12 | -1.37 | 0.93  (0.83-0.97) |
| ***3-chamber view*** | | | | | | |
| MAD, presence | 1 | 1 | - | - | - | - |
| MAD, mm | 0.93 |  | 0.10 | 0.58 | -0.38 | 0.91  (0.76-0.96) |
| ***2-chamber view, anterior wall*** | | | | | | |
| MAD, presence | 0.44 | 0.44 | - | - | - | - |
| MAD, mm | 0.76 |  | -0.09 | 0.87 | -1.04 | 0.85 (0.63-0.94) |
| ***2-chamber, inferior wall*** | | | | | | |
| MAD, presence | 0.90 | 0.90 | - | - | - | - |
| MAD, mm | 0.83 |  | 0.05 | 1.23 | -1.13 | 0.91  (0.77-0.96) |
| ***4-chamber view*** | | | | | | |
| MAD, presence | 0.90 | 0.89 | - | - | - | - |
| MAD, mm | 0.92 |  | 0.09 | 0.90 | -0.72 | 0.96  (0.90-0.98) |

MAD: Mitral Annulus Disjunction.

**Supplementary Table 6. Inter-observer agreement on MAD presence and MAD extent in the same view per single patient.**

|  | **Pearson** | **Cohen’s K Coefficient** | **Bias** | **Upper LOA** | **Lower LOA** | **ICC** |
| --- | --- | --- | --- | --- | --- | --- |
| ***All views*** | | | | | | |
| MAD presence | 0.70 | 0.78 | - | - | - | - |
| MAD, mm | 0.62 |  | -0.25 | 1.90 | -2.40 | 0.77  (0.42-0.91) |
| ***3-chamber view*** | | | | | | |
| MAD, presence | 0.79 | 0.77 | - | - | - | - |
| MAD, mm | 0.77 |  | -0.15 | 0.78 | -1.08 | 0.84  (0.60-0.94) |
| ***2-chamber view, anterior wall*** | | | | | | |
| MAD, presence | 0.69 | 0.64 | - | - | - | - |
| MAD, mm | 0.71 |  | -0.05 | 1.24 | -1.24 | 0.80  (0.49-0.92) |
| ***2-chamber view, inferior wall*** | | | | | | |
| MAD, presence | 0.53 | 0.51 |  |  |  |  |
| MAD, mm | 0.55 |  | -0.24 | 1.75 | -2.22 | 0.71  (0.27-0.88) |
| ***4-chamber view*** | | | | | | |
| MAD, presence | 0.80 | 0.78 |  |  |  |  |
| MAD, mm | 0.66 |  | 0.06 | 1.90 | -1.79 | 0.80  (0.49-0.92) |

MAD: Mitral Annulus Disjunction.

**Supplementary Table 7. MAD presence and extent in 16 subjects with both CMR and TTE available.**

|  | **MAD extent CMR, mm** | **MAD extent TTE, mm** |
| --- | --- | --- |
| **MAD+ CMR, %** | | |
| 9/16, 56% | 2.3 (1.4-6) | - |
| **MAD+ TTE, %** | | |
| 4/16, 25% |  | 4.5 (3.3-6) |
| **MAD+ CMR MAD- TTE, %** | | |
| 5/16, 31% | 1.5 (1.2-2.5) | - |
| **MAD- CMR MAD+ TTE, %** | | |
| 0/16 | - | - |
| **MAD+ CMR MAD+ TTE, %** | | |
| 4/16, 25% | 6 (4.2-6.4) | 4.5 (3.3-6) |
| **MAD- CMR MAD- TTE, %** | | |
| 7/16, 44% | - | - |

CMR: cardiovascular magnetic resonance, TTE: trans-thoracic echocardiography

**Supplementary Table 8. Firth and Poisson univariable regression analysis for the composite endpoint.**

| **Variables** | **Firth regression analysis** | | | **Poisson regression analysis** | | |
| --- | --- | --- | --- | --- | --- | --- |
|  | **OR** | **95% CI** | **P value** | **Coefficient** | **95% CI** | **P value** |
| MAD ≥1 mm | 0.63 | 0.19; 2.05 | 0.44 | -0.50 | -1.73; 0.73 | 0.42 |
| MAD ≥4 mm | 0.84 | 0.15; 4.76 | 0.85 | -0.51 | -2.57; 1.54 | 0.63 |
| MAD ≥6 mm | 4.10 | 0.69; 24.56 | 0.12 | 1.04 | -1.01; 3.10 | 0.32 |
| MVP, presence | 3.86 | 0.91; 16.39 | 0.07 | 1.15 | -0.38; 2.68 | 0.14 |

MAD: Mitral-Annulus-Disjunction; MVP: Mitral-Valve-Prolapse

**III. Supplementary Figures**

**Supplementary Figure 1. Relationship between MAD extent and MVP extent.**

A positive correlation between the extent of MAD and MVP (both expressed as mm) is observed. (r= 0.48; P=0.006).

MAD: mitral annulus disjunction; MVP: mitral valve prolapse.

**Supplementary Figure 2. Relationship between extent of MAD and mitral annulus diameter measured in systole.**

A positive correlation between the extent of MAD and mitral annulus (both expressed as mm) is observed (r= 0.34; P<0.001).

**Supplementary Figure 3**. **Bland-Altman analysis on intra-operator (A) and inter-operator (B) agreement on MAD extent measurements.**

**Supplementary Figure 3.a**

**Supplementary Figure 3.b**
